# *Toxocariasis* affecting brain stem and skull base

**DOI:** 10.1186/s12879-021-06867-1

**Published:** 2021-12-11

**Authors:** Manuel Gomez Serrano, Rafael Jimenez Rodriguez-Madridejos, Salome Merino Menendez, Diana Maria Hernanperez Hidalgo, Jesus Gimeno Hernández, Maria Cruz Iglesias Moreno

**Affiliations:** 1 ENT Department, Hospital Clínico San Carlos, Universidad Complutense, Calle Profesor Martin Lagos s/n. 5ª Planta, Madrid, Spain; 2 Internal Medicine Consultant. Internal Medicine Department, Hospital Clínico San Carlos, Universidad Complutense, Madrid, Spain; 3 Radiology Department, Hospital Clínico San Carlos, Universidad Complutense, Madrid, Spain

## Abstract

**Background:**

Toxocariasis is a helminthic infection caused by a nematode that mainly affects populations in tropical and subtropical latitudes. Humans are potential paratenic hosts, and clinical disease occurs as a result of parasite migration through intestinal tissue. We present a clinical case of otorhinolaryngological affectation by Toxocara canis.

**Case presentation:**

A 60-year-old male from Ecuador, resident in Spain for 5 years, evaluated in the emergency department for presenting headache, otorrhea and left ear pain. Computed tomography (CT) and magnetic resonance imaging (MRI) reported a large mass of the nasopharynx with infiltration of the skull base, intracranial extension and a lesion in the left pons without being able to exclude metastases. Two Functional Endoscopic Sinus Surgery (FESS) biopsies were negative for malignancy. Despite not meeting the diagnostic criteria established by the existing literature, the clinical and radiological presentation, the presence of risk factors, a positive serology for Toxocara canis (IgGELISA) and the absence of alternative diagnosis were considered sufficient criteria to establish toxocariasis with inflammatory lesions in the nasopharynx and pons as the most probable diagnosis. Treatment with albendazole (400 mg / 12 h) and corticosteroids (1 mg / kg for 5 days) was started and continued for one month. Post treatment negative serology, and MRI and CT post treatment controls were performed after one year, both showing a decrease in lesion of the clivus as well as the pons.

**Conclusions:**

With the appropriate personal history, toxocariasis should be included in the differential diagnosis of infiltrating lesions of the skull base with a negative study of tumor histology. Albendazole treatment has been shown to control and cure the disease.

## Background

Human toxocariasis is a helminthic infection caused by a nematode that primarily impacts populations in tropical and subtropical latitudes. Two main species can affect humans: Toxocara canis and Toxocara cati. The hosts (dogs, foxes, cats…) carry the nematodes in their gut, liberating eggs in feces that can be infectious for many years outside any host. After the ingestion of the eggs, the host can be of two types: final, allowing the parasite to persist in the host´s gut, and paratenic, in which the larvae can migrate to different organs to encyst. Humans are possible intermediate animal hosts and the clinical disease happens as a result of the migration of the parasite through extra-intestinal tissue. Although it is well known that it leads to severe complications such as blindness or meningoencephalitis, it usually does not cause any clinical manifestation [1, 2]. We hereby present the only known case in the English literature of otorhinolaryngological involvement by Toxocara canis.

## Case presentation

A 60-year-old male from Ecuador residing in Spain for 5 years with a personal history of liver cirrhosis of enolic origin with portal hypertension; esophageal varices and episodes of hepatic encephalopathy. At the time of his admission, his prescribed medication comprised Vidagliptin and glimepiride.

**Fig. 1:**
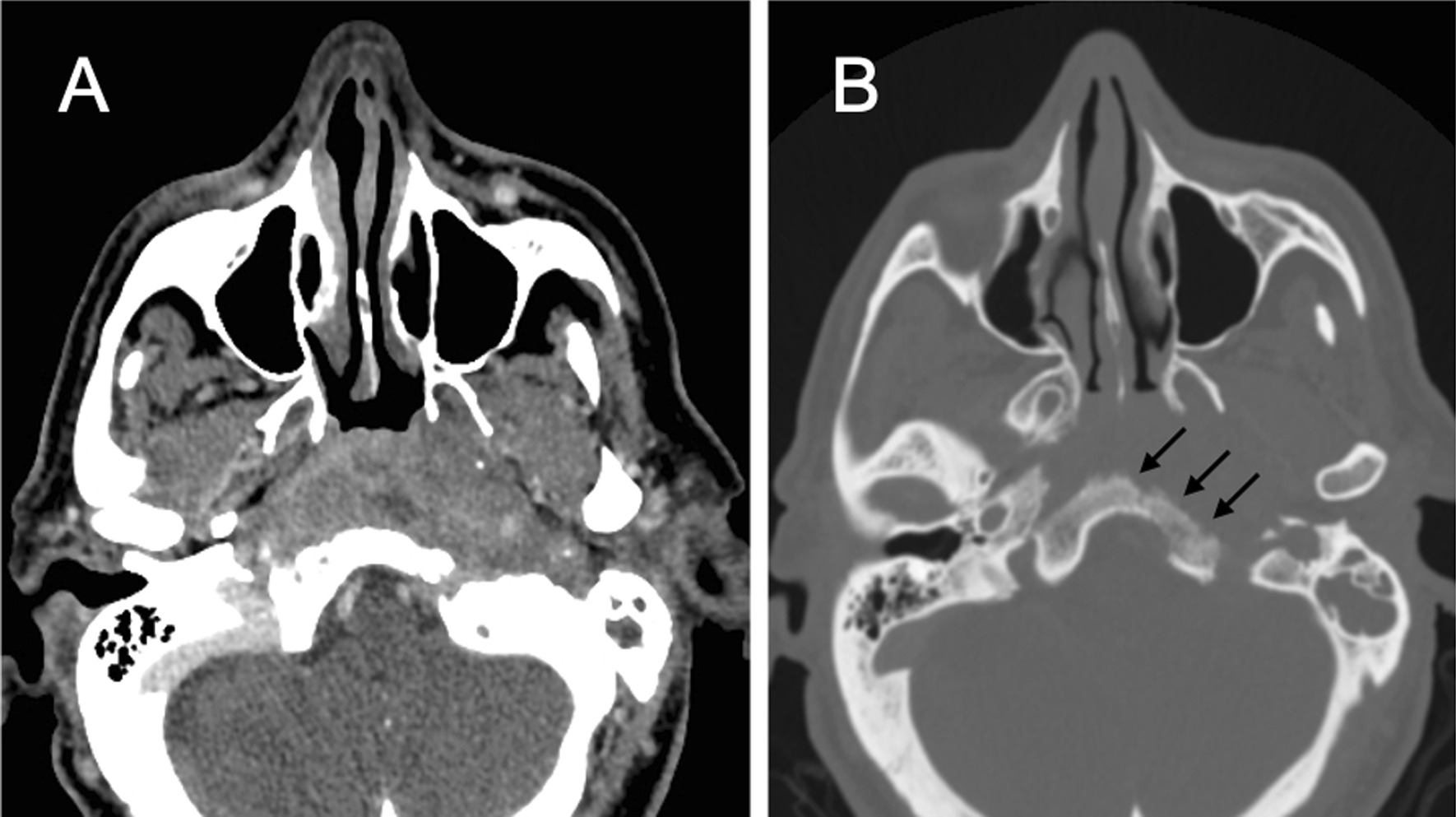
CT scan. A huge ill-defined soft tissue mass in the nasopharynx and perivascular space was seen in the midline on postcontrast CT scan. Heterogenous enhancement of the lesion (**a**) and associated bone erosion (**b**, *black arrows*) were observed. Occupation of mastoid cells on the left side were also noted

**Fig. 2:**
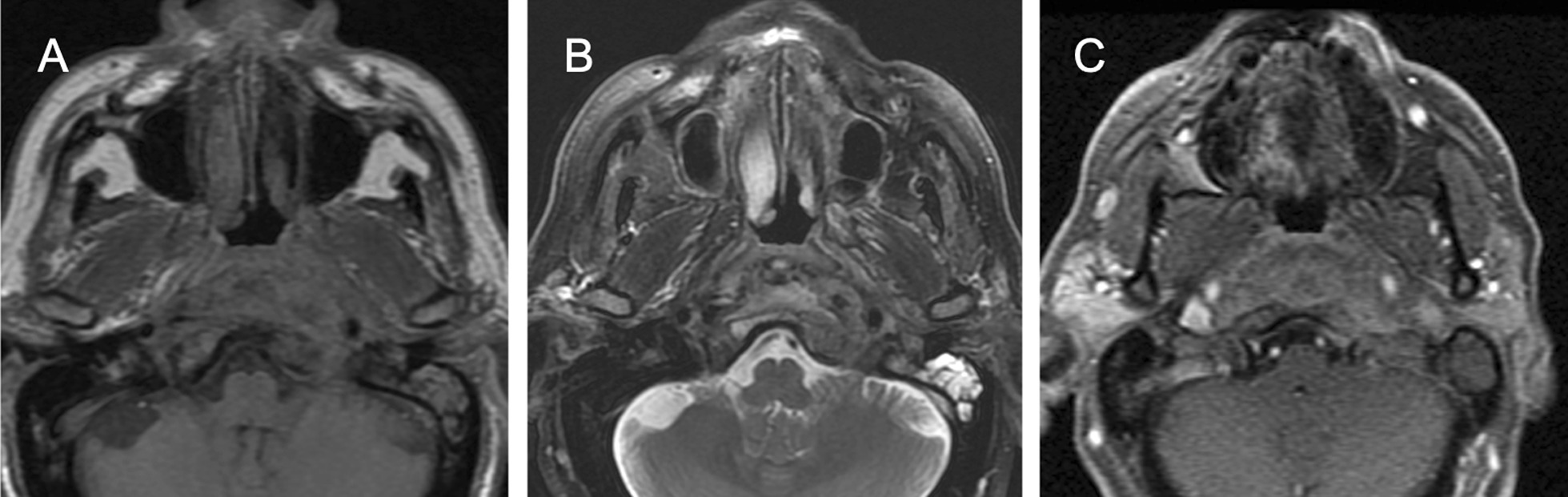
MR images obtained one week later confirmed the presence of an infiltrative hypointense on T1-weighted (**a**) and T2-weighted (**b**) images mass with irregular enhancement on post-contrast images (D). Infiltration of adjacent bone marrow is also observed

The patient was referred to outpatient Ear, Nose and Throat (ENT) as presenting left headache, otorrhea and otalgia. Examination revealed a polypoid lesion occupying the left external auditory canal (EAC), left vocal cord paralysis and normal nasal and sinus examination, including the nasopharynx. Computed tomography (CT) reported a large nasopharyngeal mass (2 × 5 cm) infiltrating the clivus (Fig. 1) with soft tissue component and heterogeneous contrast enhancement. Left ear images were compatible with chronic otitis media with no signs of cholesteatoma. A magnetic resonance imaging (MRI) scan confirmed the presence of an infiltrative mass hypointense on T1-weighted and T2-weighted with infiltration of adjacent bone marrow. Our first possible diagnosis (Fig. 2) was to consider the possibility of a tumor.

A first biopsy through functional endoscopic sinus surgery (FESS) was performed reporting a mixed inflammatory infiltrate, without any microorganisms or malignancy.

**Fig. 3:**
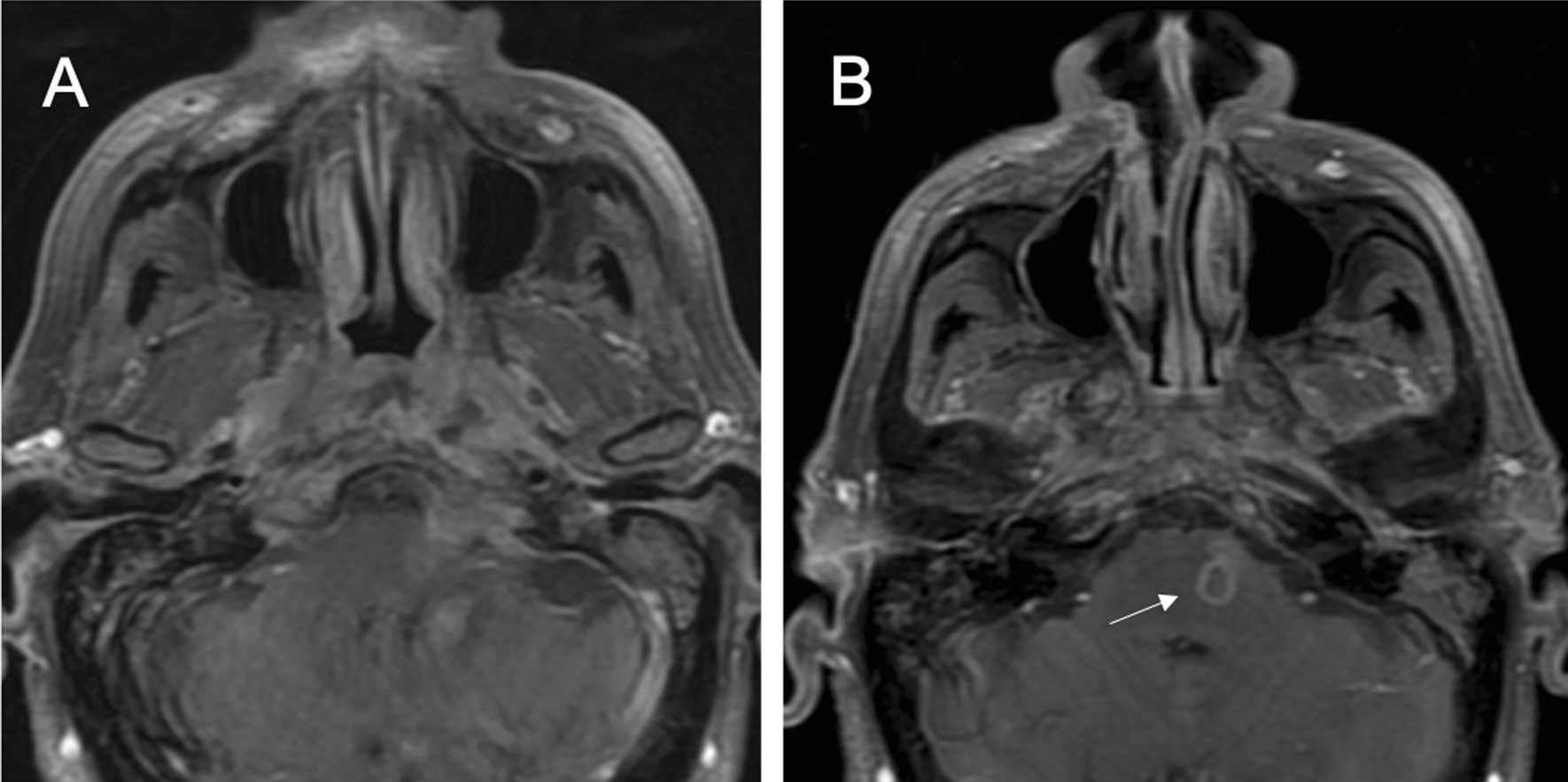
A second MR study performed one month later showed persistence of the lesion previously observed in the skull base. Extradural extension of the lesion (**a**) and an intra-axial lesion located on the left side of the pons with peripheral enhancement (**b**, *white arrow*) were seen on post- contrast T1-weighted images

**Fig. 4:**
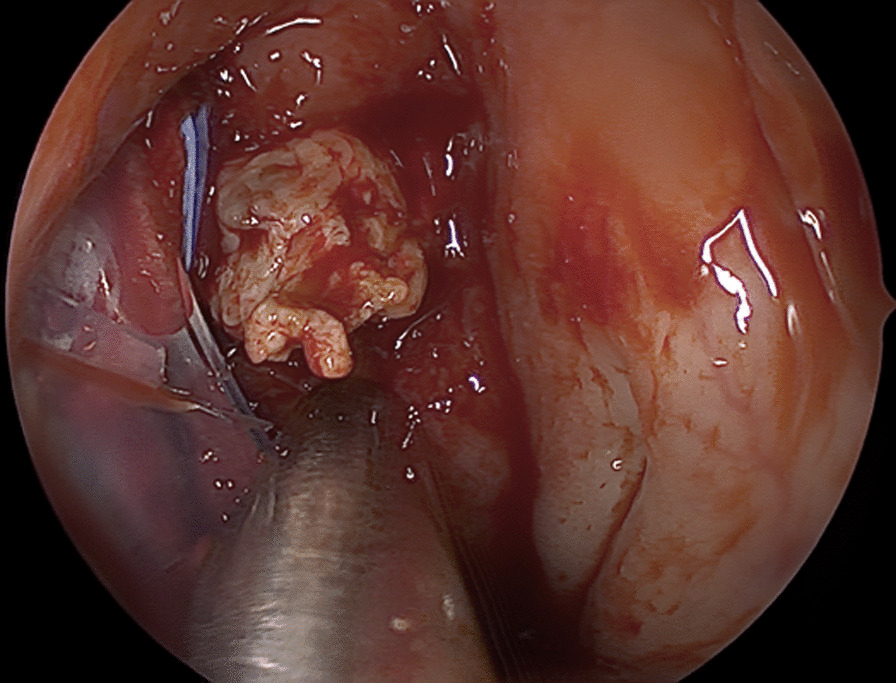
Lesion of the nasopharynx being biopsied for a second time showing hard fibrous tissue with withiest appearance

Fifteen days later the patient was admitted to the internal medicine service with the diagnosis of hydropic decompensation. A second MRI scan was performed, revealing signs of hepatic encephalopathy, progression of the nasopharyngeal mass with intracranial extension and a new lesion in the left pons, not being able to rule out metastases (Fig. 3). A second biopsy was performed finding a hard-fibrous tissue with withiest appearance (Fig. 4). Histology informed of a connective tissue with acute inflammation without malignancy data. The microbiology study was negative whilst the positron emission tomography scan and a computed tomography scan (PET-CT) revealed small non-specific left lateral cervical lymphadenopathies. Several serologies for different microorganisms were extracted, obtaining a positive result for Toxocara canis immunoglobulin G enzyme-linked Immunoassay (IgG-ELISA) (Table 1).

**Table 1:** Microbiological studies performed: Serological studies requested for differential diagnosis are presented.

Serology	26/11/2018
Syphilis (Total antibodies)	Negative
Toxoplasmosis (IgG)	Positive
Toxoplasmosis (IgM)	Negative
Borrelia burgdorferi (IgG + IgM)	Negative
Coxiella burnetii (IgG + IgM)	Negative
Rickettsia conorii (IgG + IgM)	Negative
Leishmania (IgG + IgM)	Negative
Bengal Rose	Negative
Brucella Agglutination	Negative
Brucella (Total antibodies)	Negative
Trypanosoma cruzi (IgG)	Negative
Echinococcus granulosus(IgG)	Negative
Schistosoma mansoni (IgG-ELISA)	Positive
Toxocara canis (IgG-ELISA)	Positive
Leptospira (IgM)	Negative
Strongyloides (IgG-ELISA)	Negative
Paul Bunnell (heterophilic antibodies)	Negative
Epstein-Barr (IgG EBNA)	Positive
VHC Antibodies	Negative
HBs Antigen	Negative
HBcore total Antibodies	Negative
Herpes 1–2 (IgG)	Positive
Herpes 1–2 (IgM)	Negative
VHA (IgM)	Negative
VHA (Total antibodies)	> 100 MUI/mL
Varicela (IgM)	Negative
Varicela (IgG)	Positive
Hepatitis E (IgM)	Negative
Hepatitis E (IgG)	Positive
Parvovirus B19 (IgG)	Positive
Parvovirus B19 (IgM)	Negative
HTLV I y II Antibodies	Negative
VIH antibodies and antigen p24	Negative
Cytomegalovirus (IgM)	Negative
Cytomegalovirus (IgG)	Positive
CMV viral load	Undetectable
Plasmodium antigenic study	Negative
Plasmodium study in peripheral blood	Negative

**Fig. 5:**
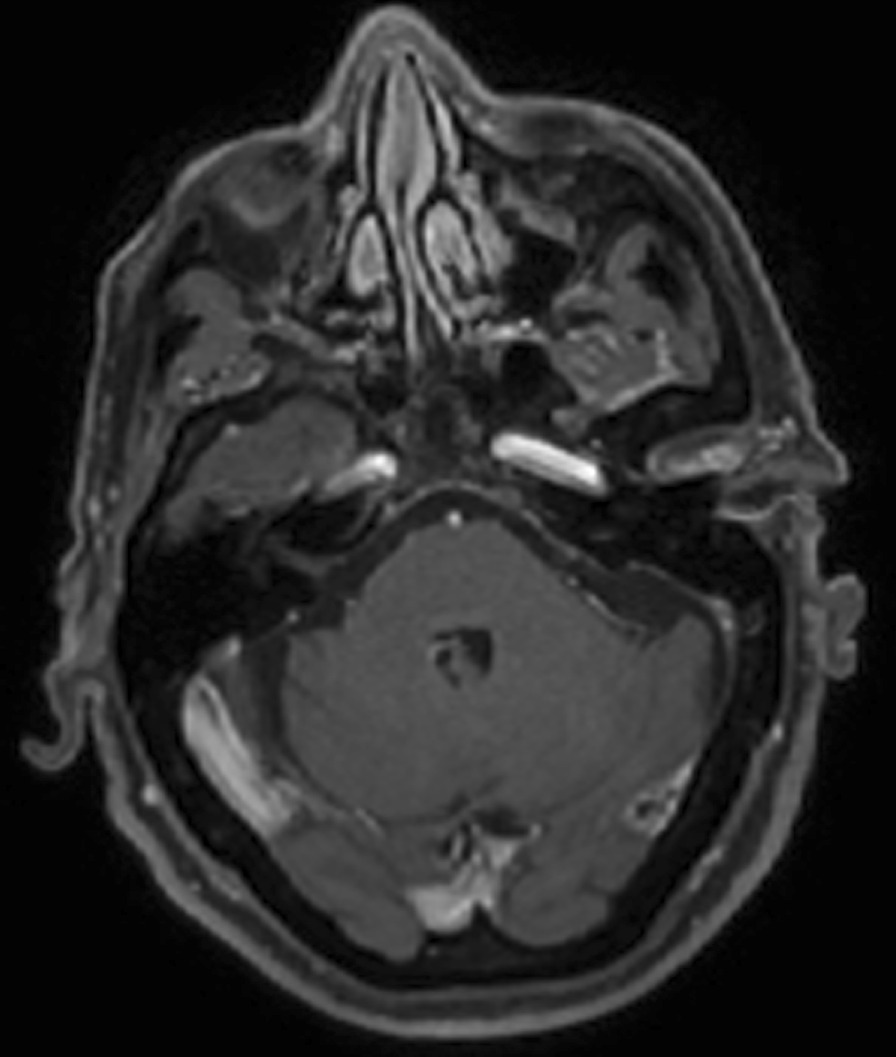
Complete resolution of radiological findings shown on previous studies were observed on MRI performed one year later. No soft tissue mass, bone infiltration or extension to posterior fossa were noted

In the absence of tumor in the histology and serological findings, toxocariasis with an inflammatory lesion in the nasopharynx and pons was established as the most likely diagnosis. The ophthalmology service ruled out any ocular involvement. Treatment with albendazole (400 mg/12 h) and corticosteroids (1 mg/kg for 5 days) was decided. During treatment, albendazole was suspended due to recurrent leukopenia. However, the patient received a total of 28 days of albendazole and, after one month of treatment, demonstrated clinical stability, negative result for Toxocara canis IgG-ELISA serology two months after the treatment was finished, and radiological improvement of soft tissue lesion of the nasopharynx and pons, persistent after a year on control MRI and CT (Fig. 5). The left vocal cord paralysis did not recover and speech therapy was indicated with good results. The chronic otitis media is currently under surveillance.

## Discussion and conclusions

Unlike the global experience in which this disease affects children in tropical and subtropical regions, we present a 60-year-old male living in Spain who had spent most of his childhood in Ecuador where he had been exposed to cats and dogs and it was probably during this time that he became infected. However, in the United States, the seroprevalence estimates a range from 5 to 15% and approximately 10,000 clinical cases are diagnosed yearly [1].

The signs and symptoms depend on the organ to which the parasitic nematode larva migrates and the host inflammatory response. In addition, the migration through the patient’s body may take several years before the nematode encysts in an organ. During this time, only persistent eosinophilia can be detected. According to the patient's personal history, he came into contact with the parasite long before the clinical manifestations (maybe even years), thus being a covert toxocariasis without eosinophilia.

There are several types described of toxocariasis (ocular larva migrans, larva migrans visceralis, covert toxocariasis, common toxocariasis, neurotoxocariasis…) [3–5]. In neurotoxocariasis, the involvement of the peripheral nervous system, reflected in radiculitis, inflammation of cranial nerves could also be developed [1, 6]. This patient presented with a large mass, involvement of the tenth cranial nerve and a lesion in the brain stem. The histological study was negative for malignancy, revealing inflammatory reaction. In the rare cases in which histological evaluation is possible, eosinophilic abscesses with surrounding granulomatous reactions are the expected manifestations. The diagnosis is difficult, the detection of the microorganism on microscopy being the gold standard. However, the proposed diagnostic criteria are a positive serology for toxocariasis together with an eosinophil count > 1000/ml or the presence of eye damage due to toxocariasis [2, 3, 7]. Specifically, the diagnosis of neurotoxocariasis is based on a body of arguments, such as a compatible clinical and radiological picture, the presence of risk factors, eosinophilia in blood and/or cerebrospinal fluid (CSF), high titers of antibodies against Toxocara spp. in blood and/or CSF, clinical and/or radiological improvement after anthelmintic therapy, and absence of any alternative diagnoses [5]. DNA detection by PCR in different tissues of experimental animals is showing promising results [7]. The diagnosis of this case is controversial and has its limitations. On the one hand, the patient does not present elevated levels of eosinophils and no ocular lesions could be demonstrated. On the other hand, the clinical and radiological picture, the presence of risk factors, the antibody titers against Toxocara spp. in blood, and the absence of alternative diagnoses were criteria to indicate treatment. Finally, clinical and radiological improvement after anthelmintic therapy reinforce the diagnostic theory of toxocariasis. Toxocara cati could not be excluded, therefore the final diagnosis is toxocariasis.

Both albendazole and mebendazole are effective. Albendazole is recommended as it has superior bioavailability than mebendazole, and a better pass through the blood–brain barrier, as well as a better tolerance. In addition, nonsteroidal anti-inflammatory drugs or corticosteroids can be administered to reduce clinical symptoms due to allergic response to the parasite’s antigens. Depending on other endemic infections, ivermectin and DEC (diethylcarbamazine) may be useful as well. Surgery is reserved for only the most severe cases [1, 7]. When neurotoxocariasis is demonstrated, the treatment should be prescribed for at least 3 weeks, and often needs to be repeated [5]. In our case, despite the toxicity of albendazole, four weeks of treatment were completed and no recurrence or progression was observed during a year of surveillance.

With the appropriate personal history, toxocariasis should be included in the differential diagnosis of infiltrating skull base lesions. To date, serology in the appropriate clinical context has been sufficient to indicate treatment with albendazole and corticoids, which have controlled and cured the disease.

## Data Availability

Data available among the clinical notes of the patient at Hospital Clinico San Carlos.
